# Recent Research Progress in Fluorescent Probes for Detection of Amyloid-β In Vivo

**DOI:** 10.3390/bios13110990

**Published:** 2023-11-19

**Authors:** Zhen-Yu Zhang, Ze-Jun Li, Ying-Hao Tang, Liang Xu, De-Teng Zhang, Tian-Yi Qin, Ya-Long Wang

**Affiliations:** 1 State Key Laboratory of Digital Medical Engineering, School of Biomedical Engineering, Hainan University, Haikou 570228, China; 2 Key Laboratory of Biomedical Engineering of Hainan Province, School of Biomedical Engineering, Hainan University, Haikou 570228, China; 3 Institute of Neuroregeneration and Neurorehabilitation, Qingdao University, Qingdao 266071, China; 4 Wuhan National Laboratory for Optoelectronics, School of Optical and Electronic Information, Huazhong University of Science and Technology, Wuhan 430074, China

**Keywords:** amyloid-β (Aβ), Alzheimer’s disease (AD), fluorescent probe, bioimaging

## Abstract

Alzheimer’s disease (AD) is a neurodegenerative disease. Due to its complex pathological mechanism, its etiology is not yet clear. As one of the main pathological markers of AD, amyloid-β (Aβ) plays an important role in the development of AD. The deposition of Aβ is not only related to the degeneration of neurons, but also can activate a series of pathological events, including the activation of astrocytes and microglia, the breakdown of the blood–brain barrier, and the change in microcirculation, which is the main cause of brain lesions and death in AD patients. Therefore, the development of efficient and reliable Aβ-specific probes is crucial for the early diagnosis and treatment of AD. This paper focuses on reviewing the application of small-molecule fluorescent probes in Aβ imaging in vivo in recent years. These probes efficiently map the presence of Aβ in vivo, providing a pathway for the early diagnosis of AD and providing enlightenment for the design of Aβ-specific probes in the future.

## 1. Introduction

Alzheimer’s disease (AD) is a degenerative disease of the central nervous system that occurs mostly in elderly people. AD is characterized by progressive cognitive dysfunction and behavioral disorders. As the most common type of dementia, it impairs the thinking, memory, and independence of patients, leading to decreased quality of life and even death. Due to its complex pathological mechanism, there is no effective treatment for AD [1,2,3,4,5]. At present, it has been reported that Leqembi and Donanemab can be used in the treatment of AD [6,7]. However, due to its complex pathological mechanism, the research on most AD therapeutic drugs is at a stagnant stage [8,9]. Relevant pathological studies have shown that the accumulation of Amyloid-β (Aβ) inside and outside the nerve cells can cause toxic reactions, leading to neuronal degeneration or death [10]. Therefore, the excessive accumulation of Aβ is considered to be an important pathological marker of AD. Hence, the detection of Aβ plays an important role in the study of AD, such as the early screening of AD, dynamically monitoring the process of AD, and evaluation of the therapeutic effect. In addition, the monitoring of Aβ can also assist in the diagnosis of craniocerebral injury, which is of great significance for judging the severity of craniocerebral injury and optimizing the treatment plan [11,12,13].

Aβ is produced by the hydrolysis of amyloid precursor protein by β- and γ-secretases. It is generally a polypeptide containing 39–43 amino acids [14,15]. Its self-assembly process forms a variety of structures with varying degrees of neurotoxicity. In humans, the most common Aβ subtypes are Aβ1–40 and Aβ1–42 [16,17]. Compared with Aβ1–40, Aβ1–42 is more inclined to aggregate, progressing from monomer to oligomer to aggregate, resulting in greater neurotoxicity [18,19]. The deposition of Aβ can cause cerebrovascular sclerosis or even rupture and induce premature apoptosis of nerve cells, which leads to corresponding pathological change [20,21].

**Figure 1:**
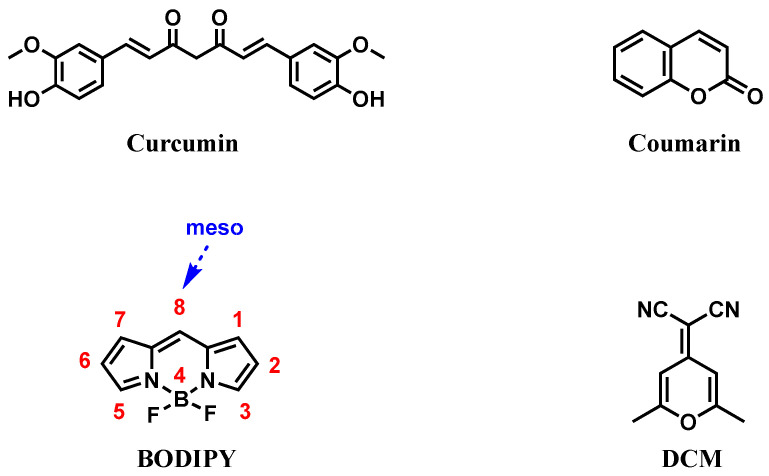
Common core structures of fluorescent probes used for Aβ detection.

At present, numerous fluorescent probes have been reported for the detection of Aβ. However, due to their inability to penetrate the blood–brain barrier (BBB), most Aβ probes can only be used for the detection of Aβ in vitro [22,23,24]. Therefore, it is of great significance to design Aβ probes that can be used for in vivo imaging [25,26]. In the development of Aβ imaging probes, many luminescent materials stand out because of their excellent photophysical properties, such as curcumin, 4,4-difluoro-4-bora-3a,4a-diaza-s-indacene (BODIPY), etc. (Figure 1) [27]. These dyes can bind to the hydrophobic cavities of Aβ, causing their fluorescence to light up and mapping the location of Aβ deposits in the brain [28,29]. It is worth mentioning that their core structures are liable to functionalize by group substitution, adjusting their photophysical properties and adapting to various conditions [30,31,32]. In recent years, these luminescent materials have been widely used in the field of Aβ imaging in vivo. Many Aβ probes have been reported one after another, but no article has systematically summarized the application of Aβ probes in vivo. Therefore, based on the structure of curcumin and other materials, we comprehensively summarized the fluorescent probes of Aβ using in vivo imaging based on core structure published in recent years (Table 1) and discussed the challenges faced by their wide application.

## 2. Fluorescent Probes In Vivo for Amyloid-β Detection

### 2.1. Curcumin-Based Probes for Aβ

Curcumin is a pigment with a diketone structure. As a typical fluorophore, it has been widely used in the field of biological imaging because of its strong luminescence and low biotoxicity [33]. Due to the extensiveness of its functionality, curcumin dyes can be considered as a unique platform for designing probes and sensors to detect and track structural changes in various Aβ aggregates. The almost unmatched versatility of curcumin dyes in terms of synthesis and dimmable light physical properties makes them one of the superior scaffolds for the development of Aβ probes. Previous studies have shown that the introduction of a difluoroboric acid group into the fluorophore of curcumin derivatives causes a red shift in its fluorescence emission [34,35]. This is because, after the boric acid group is coordinated, the empty orbital electron of oxygen is lifted to the boron atom through the π→π transition [36]. Based on these studies, difluoroboric acid is often introduced into curcumin scaffolds to synthesize new fluorophores.

**Figure 2:**
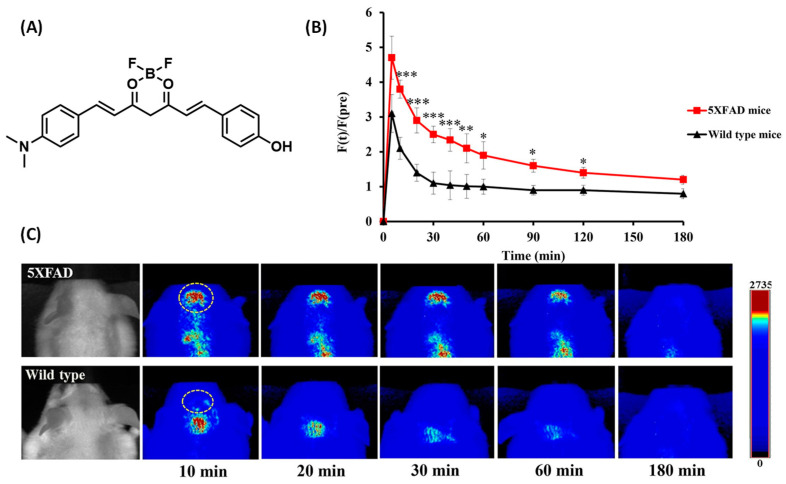
(**A**) Structure of 8b. (**B**) Comparison of fluorescent signal intensities in the brain (* *p* < 0.05, ** *p* < 0.01, and *** *p* < 0.001). (**C**) 5 × FAD mice and the control littermates at different times after the intravenous injection of 8b. (Reproduced with permission from [37], Copyright 2021, Elsevier).

Park et al. reported a near-infrared probe 8b based on the curcumin D-A-D structure. The probe uses a curcumin scaffold complexed with difluoroboronic acid as an electron acceptor, and the common N, N-dimethylamino and phenylhydroxy groups are used as electron donors at both ends (Figure 2A). Fluorophores have desirable optical properties for brain imaging in vivo. Probe 8b emits strong fluorescence at 667 nm after binding with Aβ fiber. The fluorescence difference before and after binding is 21.4 times, and the detection limit is 91.2 nm. It is worth noting that, after injecting probe 8b into the tail vein of 5 × FAD mice, the largest fluorescence signal was detected in the brains of the mice 10 min later, and the fluorescence signal reached 2.26 times that in wild mice; furthermore, the fluorescence intensity decreased significantly after 60 min (Figure 2C). In addition, the probe’s biological distribution in the brain suggests that 8b can bind to Aβ in the brains of 5 × FAD mice for more than 60 min. Moreover, it showed strong contrast between 5 × FAD mice and WT mice between 30 and 60 min. The design of the probe demonstrated the feasibility of curcumin derivatives in vivo imaging of Aβ [37].

**Figure 3:**
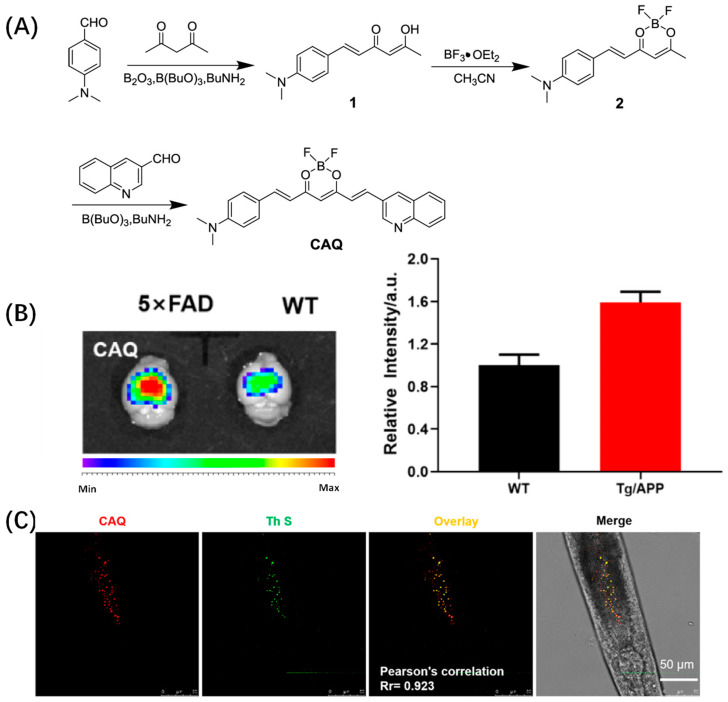
(**A**) Synthesis routes of the compounds CAQ. (**B**) Ex vivo fluorescence images of the brains of 5 × FAD transgenic mouse and WT control mouse after intravenous injection of CAQ at time point of 30 min. (**C**) Fluorescence image of AD model of *C. elegans* after incubation with ThS and CAQ for 30 min, Pearson correlation coefficient = 0.923. (Reproduced with permission from [38], Copyright 2021, American Chemical Society).

**Figure 4:**
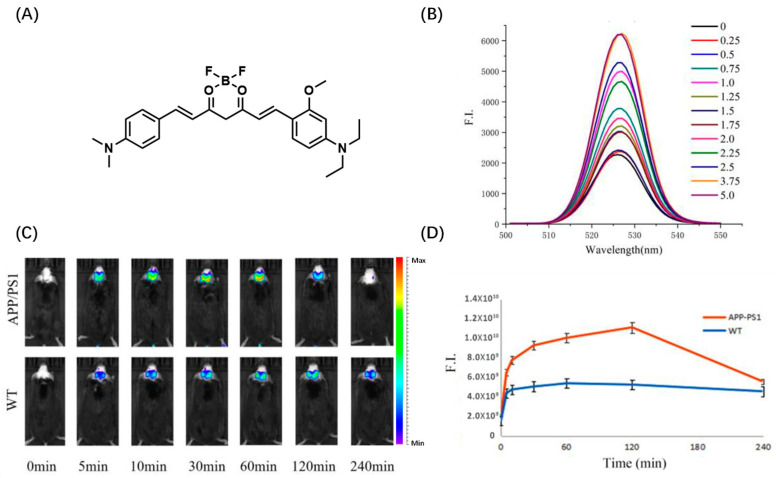
(**A**) Structure of 3b. (**B**) Fluorescence spectra of 3b combined with Aβ1–40 aggregates; concentration unit: ×10^−5^ mol/L. (**C**) Fluorescence imaging of probe 3b in vivo. (**D**) Fluorescence intensity change curve. (Reproduced with permission from [39], Copyright 2023, Elsevier).

Based on the same electron acceptor, Wu et al. changed the benzene hydroxyl terminal of the 8b electron donor into quinoline and designed Aβ probe CAQ for in vivo imaging of Aβ (Figure 3A). CAQ has obvious specificity to Aβ: the fluorescence is obvious after binding with Aβ and the red fluorescence is gradually enhanced with the increase in Aβ concentration. Later, in vivo experiments found that CAQ could enter organisms better than traditional Aβ dyes (Figure 3B). Subsequently, Wu’s team further investigated the imaging behavior of CAQ in the nematode AD model. After CAQ staining, the red fluorescence in the ganglia of *C. elegans* was significantly enhanced, indicating that CAQ has good imaging ability in vivo (Figure 3C). MTT experiment also showed that CAQ had good biocompatibility [38]. Li et al. used a similar molecular design strategy, except that the quinoline end of CAQ was changed to N, N-diethyl-3-methoxyaniline and reported a curcumin-based NIR probe 3b (Figure 4A). Probe 3b has a high affinity with Aβ1–40 (KD = 2.12 ± 0.77 μM), and the fluorescence intensity of probe 3b increased sharply after binding with Aβ1–40 (Figure 4B). The maximum emission wavelength of probe 3b is about 667 nm. Then, 120 min after administration, the fluorescence signal intensity in the brain of the APP/PS1 mouse model was more than twice that of the wild control group, which could show the pathological changes in Aβ. During in vivo imaging experiments, 3b exhibited slower brain clearance, significantly different fluorescence from wild mice, and showed a longer detection window (Figure 4C) [39].

**Figure 5:**
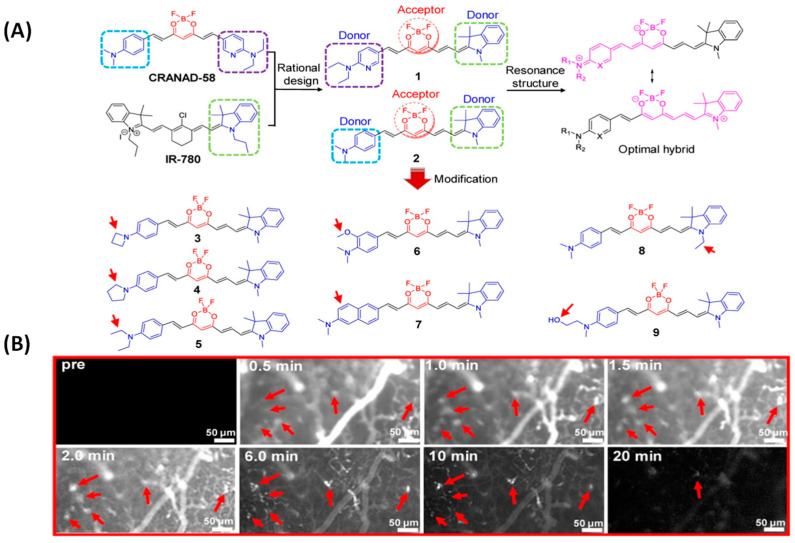
(**A**) Probes 1–9 modified by curcumin analogs. (**B**) Probe 9 was used for dynamic fluorescence imaging in 14-month-old APP/PS1 mice under an upright microscope (Nikon NIR Apo). (Reproduced with permission from [40], Copyright 2022, Ivyspring International).

In 2022, Fang et al. developed nine NIR probes for in vivo Aβ imaging by modifying curcumin analogs’ donor-acceptor-donor structure (Figure 5A). Among them, probe 9 can not only accurately distinguish AD mice from wild mice, but can also show different fluorescence intensity in AD mice at different months of age, which has certain potential in distinguishing different degrees of AD conditions. It may be that the introduction of a hydroxyl group makes probe 9 obtain more suitable lipophilicity. In addition, dynamic fluorescence imaging of AD mice with thinner skulls under an upright microscope (Nikon NIR Apo) showed that probe 9 can rapidly cross the blood–brain barrier and selectively label Aβ plaques in the brain lesion area (Figure 5B). Notably, the presence of probe 9 attenuated Aβ aggregation [40].

The summary of aforementioned probes has validated the potential of Aβ detection using curcumin derivatives. However, it is worth noting that the emission wavelength of curcumin itself is limited to the blue region. After introduction of the difluoroboric group, the emission of curcumin derivatives significantly red-shifts to the near-infrared region. Additionally, the fluorescence enhancement of curcumin probes upon binding to Aβ is not significant, which results in low signal-to-noise ratio (SNR). Therefore, it is recommended to optimize these challenges and develop Aβ probes with high SNR in the future.

### 2.2. Coumarin-Based Probes for Aβ

Coumarin and its derivatives are UV-excited blue fluorescent dyes with an emission wavelength of about 390–480 nm. It is often used in the preparation of blue fluorescent peptides and other biomolecules [41]. Compared with some traditional fluorophores, coumarins are widely used in optical imaging because of their high fluorescence quantum yield, tunable photophysical and photochemical properties, and good stability [42,43].

**Figure 6:**
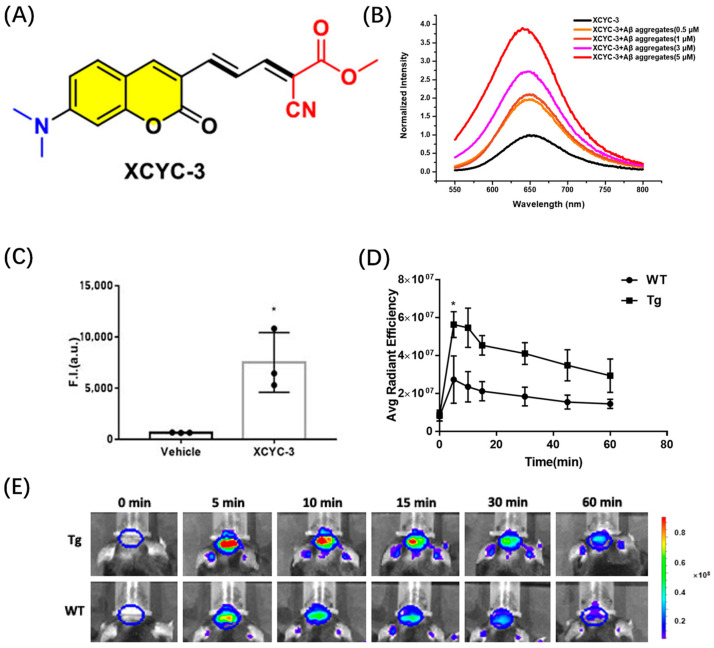
(**A**) Structure of XCYC-3. (**B**) Fluorescence intensity of XCYC-3 with different concentrations of Aβ1−42 aggregates. (**C**) The BBB penetration ability test of XCYC-3 (* *p* < 0.05). (**D**) Comparison of fluorescent signals in the brain of 5 × FAD mice and WT mice after intravenous injection. (**E**) Fluorescence imaging of brain regions in 5 × FAD and WT mice at specific time points before and after intravenous injection. (Reproduced with permission from [44], Copyright 2023, American Chemical Society).

Cao et al. designed an efficient Aβ recognition fluorescence probe XCYX-3 by embedding an aromatic coumarin framework into the π bridge of the push–pull chromophore (Figure 6A). XCYC-3 can effectively distinguish between Aβ aggregates and Aβ monomers. The fluorescence intensity of the probe was significantly enhanced after it was combined with Aβ fiber (Figure 6B). Cytotoxicity tests showed that probe XCYX-3 had good biocompatibility. In the test of blood–brain barrier permeability, the fluorescence intensity in the brains of mice injected with XCYC-3 was significantly higher than that in mice not injected with the probe, indicating that XCYX-3 had good blood–brain barrier penetration (Figure 6C). Then, the in vivo Aβ imaging ability of XCYC-3 was evaluated by detecting the fluorescence changes in the brain region of AD mice; 5 min after intravenous injection of XCYC-3, the fluorescence intensity in the brain of 5 × FAD-transgenic mice was significantly higher than that in age-matched wild-type (WT) mice (Figure 6E) [44]. 

These results indicate that coumarin-based probes are promising fluorescent probes for Aβ in vivo. However, the absorption and emission wavelengths of coumarin are relatively short, resulting in limited tissue penetration depth, which may affect the in vivo imaging effect. Additionally, the unsaturated lactone structure of coumarin dyes is prone to hydrolysis under alkaline conditions. This instability poses a challenge in the in vivo imaging of Aβ.

### 2.3. BODIPY-Based Probes for Aβ

The BODIPY series dyes have been well studied and have attracted much attention due to their special structure and versatility [45]. Compared with other traditional small molecule probes, BODIPY fluorescent dyes have excellent photochemical and photophysical properties. These mainly include (1) higher molar extinction coefficient, which is conducive to the photosensitivity of dyes; (2) high fluorescence quantum yield—the fluorescence quenching is not easy, and it can be applied to biological analysis in a variety of environments; (3) good light stability and strong anti-interference ability; (4) small peak width of the fluorescence spectrum and high detection sensitivity [46,47]. These excellent properties make the application of this kind of dye develop rapidly, and it has become the focus of the fluorescent dye [48].

**Figure 7:**
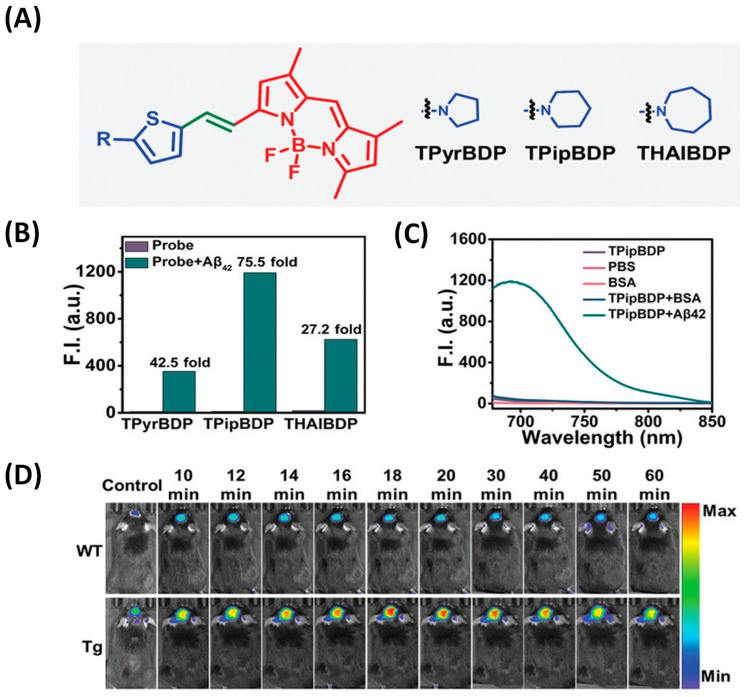
(**A**) Probe structure. (**B**) Fluorescence response of probe to Aβ aggregates. (**C**) Fluorescence response of TPipBDP to Aβ1–42 aggregates and BSA. (**D**) In vivo fluorescence imaging of brain regions in wild-type mice and APP/PS1 mice. (Reproduced with permission from [49], Copyright 2023, Wiley-VCH).

Ma et al. proposed a BODIPY-based D-π-A structure probe design strategy. In this work, Ma’s team used cyclic amines instead of common N, N-dimethylamines as electron donors to synthesize three probes (Figure 7A). Because cyclic amines have a more rigid structure, the rigid planar structure is conducive to improving the fluorescence intensity and response to Aβ of the probe. Among the three probes, TPipBDP showed the best response to Aβ1–42 aggregates: the fluorescence intensity was increased by 75.5 times and the KD value indicated that the probe had a high affinity for Aβ (Figure 7B). The fluorescence response of bovine serum albumin (BSA) to the probe may result in non-specific fluorescence enhancement, thus generating a false signal. Therefore, the interaction between the probe and BSA was evaluated, and the results showed that the presence of BSA did not enhance the fluorescent signal marks of TPipBDP (Figure 7C). In the follow-up in vivo imaging, the fluorescence signal in the brain of APP/PS1 mice was significantly higher than that in WT mice and the maximum fluorescence intensity was about three times that in WT mice (Figure 7D) [49].

**Figure 8:**
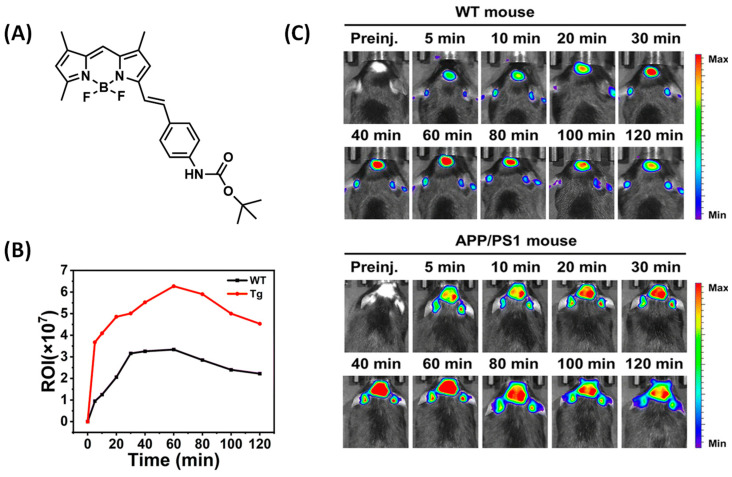
(**A**) Structure of BocBDP. (**B**) Relative fluorescence signals in brain regions of WT mice and Tg mice at different time points. (**C**) After intravenous injection of BocBDP, fluorescence imaging of brain regions in WT mice and APP/PS1 mice was performed at different time points. (Reproduced with permission from [50], Copyright 2023, Royal Society of Chemistry).

Zhu et al. designed a small-molecular probe BocBDP with D-π-A structure by using a tert-butylcarbonyl (Boc) modified aniline unit as an electron donor and a BODIPY unit as an electron acceptor through π-bridge coupling (Figure 8A). The hydrogen bond interaction between the carbonyl oxygen atom and Lys16 in the Boc unit facilitates binding to Aβ and stable imaging over long periods of time. It is worth mentioning that BocBDP has suitable lipophilicity, allowing it to cross the blood–brain barrier and maintain imaging of Aβ in the brain for more than two hours (Figure 8C) [50].

**Figure 9:**
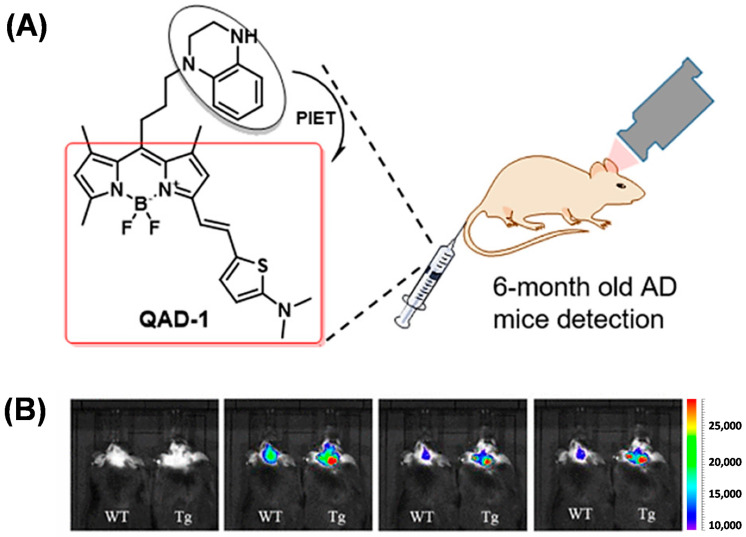
(**A**) Structure of QAD-1. (**B**) Comparison of intracranial fluorescence in QAD-1 imaging APP/PS1 mice and C57 mice. (Reproduced with permission from [51], Copyright 2018, American Chemical Society).

In addition, Ren et al. designed the probe QAD-1 based on the photoinduced electron transfer quenching mechanism, which uses BODIPY as the fluorophore and tetrahydroquinoline as the quenching group (Figure 9A). QAD-1 showed a distinct fluorescent switch after binding to both soluble and insoluble Aβ. In vivo imaging showed that QAD-1 accurately detected the presence of Aβ in APP/PS1 mice at 6 months of age. QAD-1 showed the advantage of low background signal and good elimination kinetics. The fluorescent signal in the mice reached its maximum at 5 min post-injection, and 90% of the probe was eliminated at 30 min (Figure 9B) [51].

**Figure 10:**
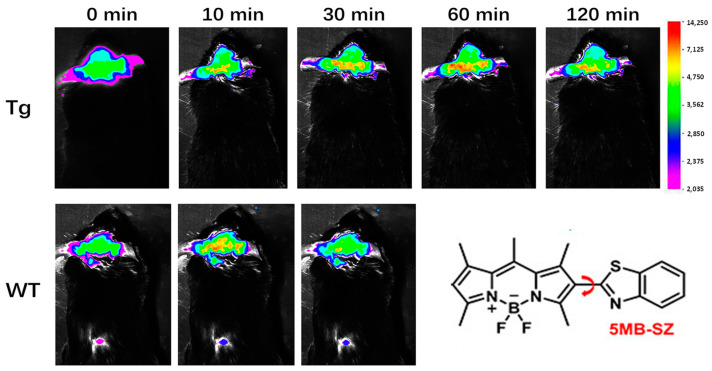
Comparison of representative fluorescence images of transgenic (APP/PS1) and wild-type (WT) mice before and after injecting 5MB-SZ into the tail vein at different time points. (Reproduced with permission from [52], Copyright 2022, American Chemical Society).

By introducing benzothiazole as a rotor at position 2 of the BODIPY core, Wang’s team prepared a probe (5MB-SZ) with an extended π-conjugate bridge. Due to the introduction of the benzothiazole group, 5MB-SZ can easily be inserted into the hydrophobic cavity in Aβ, which limits the free rotation of the single bond leading to a sharp increase in fluorescence intensity. When combined with Aβ oligomer, the fluorescence intensity of 5MB-SZ is increased by 43.64 times, and the fluorescence quantum yield is increased from 0.85% to 27.43%. After intravenous administration of 5MB-SZ, the fluorescence signals of APP/PS1 and WT mouse brain sections were significantly enhanced at 2 min, confirming that 5MB-SZ could easily penetrate the blood–brain barrier. After 10 min, the fluorescence signals of the APP/PS1 group and WT group were significantly different; the fluorescence signal of the WT group was gradually weakened, while that of the APP/PS1 group was gradually enhanced, indicating that 5MB-SZ specifically bound Aβ (Figure 10) [52]. 

These probes utilize BODIPY as the core skeleton and introduce different functional groups to design various Aβ probes. However, most BODIPY dyes demonstrate poor water solubility, leading to strong background fluorescence, which limits their applications in aqueous environments and biological systems. Therefore, finding a balance between the hydrophilicity of BODIPY dyes is a critical challenge in the development of such probes.

### 2.4. DCM-Based Probes for Aβ

As one of the typical fluorophores, dicyanomethylene-4H-pyran (DCM) is widely used in biosensing, target detection, and other fields due to its excellent photophysical and photochemical properties [53]. The mechanism of DCM derivatives’ luminescence is mainly intramolecular charge transfer (ICT), so the probe can act specifically on different target molecules by adjusting the structure of the donor and the receptor, as well as the distance.

**Figure 11:**
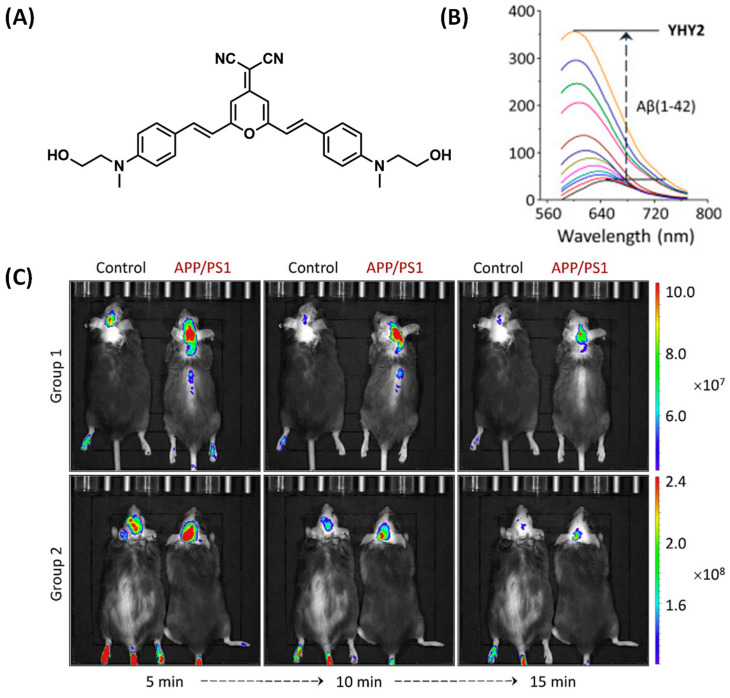
(**A**) Structure of YHY2. (**B**) Fluorescence changes of YHY2 in the presence of Aβ1–42. (**C**) Imaging comparison between 18-month-old APP/PS1 living mice and wild-type mice after tail vein injection of YHY2. (Reproduced with permission from [54], Copyright 2017, Elsevier).

Based on DCM, Yang et al. developed a D-A-D-structured fluorescent probe, YHY2, which was designed with a tertiary amine as the electron donor and malononitrile as the electron acceptor (Figure 11A). YHY2 can be used to rapidly detect Aβ peptide monomers, oligomers, and fibrils in aqueous solutions. Furthermore, it can also quickly stain Aβ depositions in the brains of transgenic mice by intravenous injection. In previous studies, the hydroxyl group of YHY2 was replaced by a methyl group, resulting in an inability to bind to Aβ. This suggests that the hydroxyethyl group of YHY2 may be critical to the binding properties of Aβ. In the presence of Aβ aggregates, the fluorescence intensity of YHY2 was significantly enhanced (Figure 11B). After incubation with mouse fibroblasts, the survival rate of the cells was more than 90%, showing good biocompatibility. During in vivo imaging experiments, APP/PS1 mice showed higher fluorescence intensity than wild-type mice for 15 min (Figure 11C) [54]. The above experiments confirm the ability of YHY2 to detect AB in vivo.

**Figure 12:**
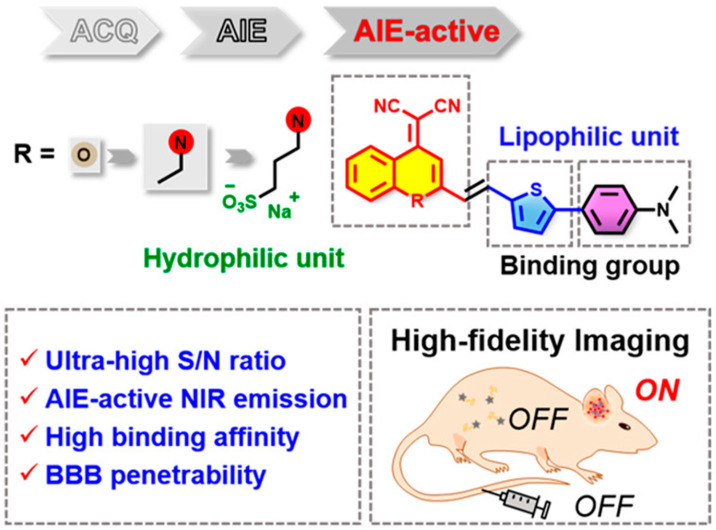
Structure and advantages of probe QM-FN-SO_3_. (Reproduced with permission from [55], Copyright 2019, American Chemical Society).

Commercially available thioflavin derivatives (ThT or ThS) have been used to detect Aβ, but past practice has shown that the ability of thioflavin derivatives to detect Aβ in vivo is very limited. Fu et al. designed a DCM-based probe QM-FN-SO_3_ by improving the defects of ThT, such as ACQ effect, low S/N ratio, and limited BBB penetrability (Figure 12B. The probe was redshifted to the near-infrared region by introducing a thiophene-conjugated π bridge. Then, quinoline malonitrile was used as the AIE building block to overcome the quenching effect. Finally, the fluorescence of the probe was turned off before binding with Aβ by adjusting the substitution position of the sulfonate group. QM-FN-SO_3_ has an extremely high signal-to-noise ratio, strong BBB penetration, and high-performance near-infrared emission characteristics, enabling high-fidelity imaging of Aβ plaques in the brain. Compared with ThS, QM-FN-SO_3_ emits more strongly during Aβ enrichment and can label and amplify fluorescence signals more accurately. During in vivo experiments, the fluorescence intensity of QM-FN-SO_3_ in the brain region of APP/PS1 mice was much higher than that in wild-type mice. In addition, according to MTT detection, the probe QM-FN-SO_3_ has good biocompatibility [55].

**Figure 13:**
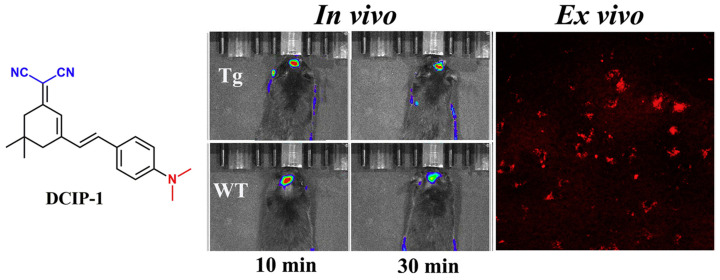
DCIP-1 was used for imaging live mice and ex vivo brain slices. (Reproduced with permission from [56], Copyright 2017, Elsevier).

Furthermore, Zhu’s team synthesized DCIP-1, using the DCM-like dicyano-isophorone chromophore, which is more selective to Aβ aggregates than other intracellular proteins. The calculated detection limit is as low as 109 nM. When combined with the hydrophobic cavity of Aβ, the probe showed a large fluorescence enhancement. In vivo imaging studies showed that the probe could penetrate the blood–brain barrier and label Ab plaques in 10-month-old transgenic live mice (APP/PS1), with fluorescence lasting more than 60 min (Figure 13) [56].

**Figure 14:**
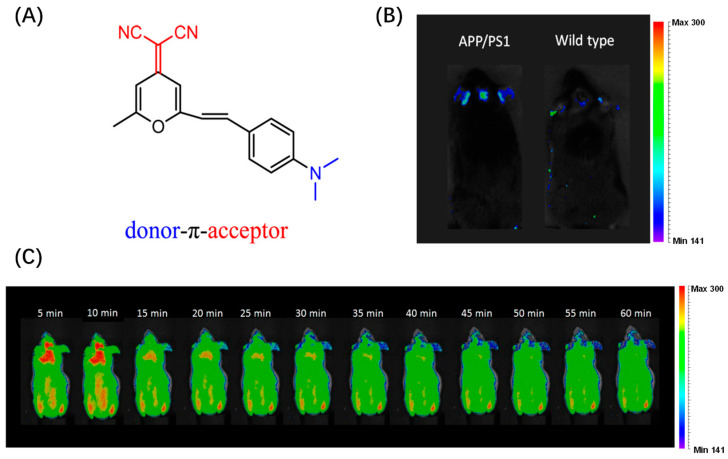
(**A**) Structure of PAD-1. (**B**) Comparison of 60 min fluorescence images of APP/PS1 transgenic mice (**left**) and wild-type mice (**right**) after intravenous injection of PAD-1. (**C**) In vivo fluorescence imaging of normal mice at different time points after intravenous injection of PAD-1. (Reproduced with permission from [57], Copyright 2015, American Chemical Society).

Based on the study of DCM, Cheng et al. designed an intelligent fluorophore PAD-1 for in vivo fluorescence imaging of Aβ in the brain (Figure 14A). PAD-1 showed a significant fluorescence enhancement effect when bound to aggregated Aβ. To evaluate the blood–brain barrier permeability of PAD-1, in vivo fluorescence experiments were performed on normal mice. After intravenous injection of the probe, the brain of the mice showed a high fluorescence signal immediately. In vivo imaging of brain signals at each time point showed that PAD-1 crossed the blood–brain barrier with high initial uptake, peaking 5 min after injection and rapidly clearing from the brain, which is highly desirable for in vivo detection of Aβ (Figure 14C). In addition, it also showed specific labeling of Aβ deposits in APP/PS1 transgenic mouse brains (Figure 14B) [57].

DCM derivatives possess numerous advantages and hold great potential for applications in biosensing and bioimaging. Similar to BODIPY dyes, most DCM probes exhibit poor water solubility and require the use of DMSO as a solvent for biological applications. However, excessive DMSO may be harmful to biological systems. Therefore, achieving appropriate hydrophilicity is a critical challenge in the further development of DCM probes.

## 3. Conclusions and Outlook

In this review, most of the imaging mechanisms of Aβ probes are based on ICT effects. By modifying the electron donor and acceptor groups in the molecular structure, the probe can obtain different luminescence properties to meet the specific tracking of Aβ in the complex brain environment. These probes have excellent photostability and can continuously monitor Aβ deposition in complex brain environments. Most probes can image Aβ in the brain for more than 60 min. They reveal the form and structural changes in the existence of Aβ in the brain, providing assistance for the pathological study of AD. But these works are still in the basic research stage and cannot be used for AD detection in humans. According to the main structure, these fluorescent probes are divided into four categories (curcumin, coumarin, BODIPY, and DCM) in this paper. With the rapid development of fluorescent probes and biosensing in recent years, these luminescent groups will also shine in the field of biosensing. 

Despite significant advances in tracking Aβ dyes in vivo in recent years, there are still major challenges: (1) NIR II has a high tissue penetration, and the design of NIR II probes is necessary; (2) It tries to balance the lipophilicity and hydrophilicity of the dye as much as possible, so that it maintains a low fluorescence background while crossing the blood–brain barrier; (3) Researchers should develop a reliable integrated probe for diagnosis and treatment, which can detect Aβ while disassembling the generated Aβ. In summary, this mini-review summarizes the excellent reports of most probes in this field, and it is foreseeable that small molecule fluorescent probes for in vivo detection of Aβ will attract increasing attention and be greatly developed. We hope that the design strategy of Aβ probes can provide some guidance for the subsequent development of similar probes. At the same time, it is also hoped that these probes will be helpful to the pathological study of AD.

## Figures and Tables

**Table 1:** Summary of properties of the probes binding with Aβ.

	Compounds	λex (nm)	λem (nm) with Aβ	(F_Aβ_/F_0_)	Kd (nM)
Curcumin	8b	560	667	21.4	91.2 ± 3.28
	CAQ	565	635	10	78.89
	3b	635	667	/	2.12 ± 0.77
	probe 9	620	697	10	14.57 ± 1.27
Coumarin	XCYX-3	502	632	/	71.11
BODIPY	TPipBDP	/	692	75.5	28.30 ± 5.94
	BocBDP	/	/	5	67.8 ± 3.18
	QAD-1	635	700	/	27
	5MB-SZ	/	550	43.64	/
DCM	YHY2	/	596	7.7	23.5
	QM-FN-SO_3_	/	/	50	170
	DCIP-1	/	635	/	674.3
	PAD-1	/	/	7	58.9

## Data Availability

No new data were created or analyzed in this study. Data sharing is not applicable to this article.
